# Association of non-selective β blockers with the mortality risk in decompensation cirrhosis: a systematic review and meta-analysis

**DOI:** 10.3389/fphar.2026.1920788

**Published:** 2026-08-05

**Authors:** Junchao Zhang, Yehong Lin, Xiaxia Weng

**Affiliations:** 1 Department of Gastroenterology, Xiamen Hospital of Traditional Chinese Medicine, Xiamen, China; 2 Department of Gastroenterology, Xiamen Hospital Dongzhimen Hospital, Beijing University of Chinese Medicine, Xiamen, China; 3 Department of Gastroenterology, Xiamen TCM Hospital Affiliated to Fujian University of Traditional Chinese Medicine, Xiamen, China; 4 Department of Infection, the First Affiliated Hospital of Xiamen University, Xiamen, China

**Keywords:** Cirrosis, decompensated, meta-analysis, morality, β-blockers

## Abstract

**Background and Aims:**

The role of non-selective beta-blockers (NSBBs) in patients with decompensated cirrhosis remains controversial, with conflicting evidence regarding their safety and efficacy across different stages of disease severity. This meta-analysis aimed to evaluate the association between NSBB use and mortality in patients with decompensated cirrhosis.

**Methods:**

We systematically searched PubMed, Embase, the Cochrane Library, and Web of Science from inception to May 2026. Studies reporting the risk of mortality associated with NSBB use in decompensated cirrhosis were included. Pooled HRs with 95% confidence intervals (CIs) were calculated using random-effects models. Subgroup analyses were performed based on mean arterial pressure (MAP), Model for End-stage Liver Disease (MELD) score, NSBB dosage, and cirrhosis complications.

**Results:**

24 studies comprising 120,072 patients were included. NSBB use was associated with a significant 25% reduction in mortality risk (HR = 0.75, 95%CI: 0.66–0.85, P < 0.01), albeit with substantial heterogeneity (I^2^ = 87%). The protective effect was consistent across studies employing propensity score matching (HR = 0.72, 95%CI: 0.59–0.88, P < 0.01) and multivariable regression analyses (HR = 0.74, 95%CI: 0.61–0.89, P < 0.01) by sensitivity analysis. Subgroup analyses revealed that the mortality benefit was more pronounced in patients with consistent baseline blood pressure (BP) between groups (HR = 0.82, 95%CI: 0.89–0.97), MELD score ≤15 (HR = 0.72, 95%CI: 0.56–0.92), and those receiving low-dose NSBBs (HR = 0.74, 95%CI:0.60–0.91). Conversely, NSBB use was not associated with reduced mortality in patients with inconsistent baseline BP (HR = 1.25, 95%CI: 0.52–3.00), MELD score >15 (HR = 0.92, 95%CI: 0.63–1.35), or high-dose NSBB therapy (HR = 0.92, 95%CI: 0.64–1.32). The presence of ascites, spontaneous bacterial peritonitis or hepatic encephalopathy appeared to not aggravate the survival risk.

**Conclusion:**

Decompensated cirrhosis should not be regarded as an absolute contraindication to NSBB therapy; rather, the decision to initiate treatment, particularly at a low dose, may be guided by MAP and MELD scores. Nonetheless, these findings require further validation through randomized controlled trials.

**Systematic Review Registration:**

https://www.crd.york.ac.uk/PROSPERO, CRD420261406986.

## 1 Introduction

Non-selective beta-blockers (NSBBs) constitute the cornerstone of pharmacological therapy for portal hypertension. By antagonizing β1-adrenergic receptor-mediated cardiac contractility and inhibiting β2-adrenergic receptor-mediated splanchnic vasodilation, NSBBs effectively lower portal pressure and have been recommended for primary and secondary prevention of variceal bleeding in patients with cirrhosis (Albillos et al., 2023; de Franchis et al., 2022). Beyond their hemodynamic effects, NSBBs may offer additional potential benefits, including the exertion of systemic anti-inflammatory activity and a reduction in the risk of further decompensation (Jachs et al., 2021; Tittanegro et al., 2022).

Nonetheless, NSBBs are not invariably beneficial in cirrhotic patients, particularly in patients with decompensated disease. As early as 2010, Sersté et al. (2010) reported that NSBBs use was associated with increased mortality in individuals with refractory ascites. Subsequently, Krag et al. (2012) proposed the “window hypothesis,” which proposes that NSBBs confer protection in the context of moderate-to-severe esophageal varices, whereas the therapeutic window of benefit closes once patients progress to a advanced stage, such as refractory ascites. Mandorfer et al. (2014) further observed that NSBBs elevate the risk of hepatorenal syndrome (HRS) and mortality in cirrhosis patients with spontaneous bacterial peritonitis (SBP). In subsequent years, however, the window hypothesis has been challenged. A *post hoc* analysis of three randomized controlled trials demonstrated that NSBBs did not increase mortality in patients with refractory ascites and, improved survival in the subgroup with mean arterial pressure (MAP) of 81–90mmHg (Bossen et al., 2016). In a propensity-matched cohort, NSBB therapy was associated with a reduced risk of death from SBP (Nawaz et al., 2026). A retrospective study (Kang et al., 2021) further revealed that, among patients with moderate or severe ascites, low-dose NSBB group exhibited a significantly greater reduction in mortality than the non-NSBB group, and the magnitude of this benefit surpassed that associated with high-dose NSBB therapy. Similarly, Lee et al. (2020) found that low dose of propranolol treatment was associated with superior survival in cirrhotic patients with hepatic encephalopathy (HE). Two meta-analyses demonstrated that NSBB therapy did not impair survival in patients with cirrhosis and ascites (Facciorusso et al., 2018; Wong et al., 2019). However, the evidence synthesized in these meta-analyses was predominantly derived from individuals with ascites and did not encompass other decompensated complications. Moreover, they did not account for the potential modifying effects of MAP, the severity of liver disease, or medication dosage on the outcomes. We therefore conducted a meta-analysis to systematically assess the impact of NSBBs on mortality in patients with decompensated cirrhosis, and performed further subgroup analyses stratified by MAP, MELD score, and drug dosage.

## 2 Methods

This meta-analysis was conducted in accordance with the guidelines for systematic reviews and meta analyses (PRISMA) statement.

### 2.1 Registration

The PROSPERO registration number for this study was CRD420261406986.

### 2.2 Search strategy and study selection

Two authors independently searched PubMed, Embase, the Cochrane Library, and Web of Science from inception through May 17, 2026,with english restrictions. The search strategy combined MeSH terms and free-text keywords. The search terms were (“Liver Cirrhosis”) AND (“Varicose Veins” OR “Hypertension, Portal” OR “Peritonitis” OR “Ascites” OR “Hepatorenal Syndrome” OR “Hepatic Encephalopathy”) AND (“Adrenergic beta-Antagonists” OR “Propranolol” OR “Nadolol” OR “Carvedilol”).

Two authors independently screened titles and abstracts, followed by full-text review of potentially eligible studies. Disagreements were resolved through consensus or consultation with a third reviewer. Studies were included if they evaluated the association between NSBB use and mortality in patients with decompensated cirrhosis. Exclusion criteria were as follows: (1) duplicate publications; (2) reviews or meta-analyses; (3) case reports; (4) letters, editorials, or commentaries; (5) conference abstracts; and (6) studies that did not pertain to the target population; (7) compared outcomes between patients treated with NSBBs vs. another agent.

### 2.3 Data extraction and quality assessment

Two investigators (JCZ and YHL) independently extracted data using a standardized data collection form. Extracted variables included: first author, publication year, country, study design, sample size, follow-up duration, patient characteristics, NSBB type and dosage, and HRs with 95% CIs for mortality.

The quality of the included cohort studies was assessed using the Newcastle–Ottawa Scale (NOS). Scores ranged from 0 to 9 stars, with ≥7 stars indicating high quality, 4–6 stars indicating moderate quality, and ≤3 stars indicating low quality. One study was a *post hoc* observational analysis derived from a randomized controlled trial; because the comparison of NSBB users versus non-users was non-randomized, it was treated as a cohort study for quality assessment and data synthesis.

### 2.4 Definition

Decompensated cirrhosis was defined by the presence of decompensation events, including ascites, SBP, HE, HRS, or jaundice. Low-dose studies were defined as those in which more than 50% of enrolled patients received a low dose of NSBB, defined as propranolol or nadolol <80 mg daily, or carvedilol ≤12.5 mg daily. Studies in which low-dose recipients accounted for <50% of the sample were classified as high-dose studies.

### 2.5 Statistical analyses

Meta-analyses were performed using Stata 14. Heterogeneity was quantified with the I^2^ statistic and assessed further by the P value from the Cochran Q test. When the P value >0.1 and I^2^ was ≤50%, heterogeneity was judged non-significant and a fixed-effects model was applied; otherwise, a random-effects model was used. To explore potential sources of heterogeneity, subgroup analyses were carried out. Sensitivity analyses were conducted by the analytical approach of the original studies, re-running the meta-analysis, and comparing the resulting estimates with the original pooled result. Publication bias was quantitatively evaluated by both Egger’s test, with a two-sided P value <0.05 considered indicative of significant asymmetry. All tests were two-tailed with a significance threshold of α = 0.05.

## 3 Results

### 3.1 Study selection

**FIGURE 1:**
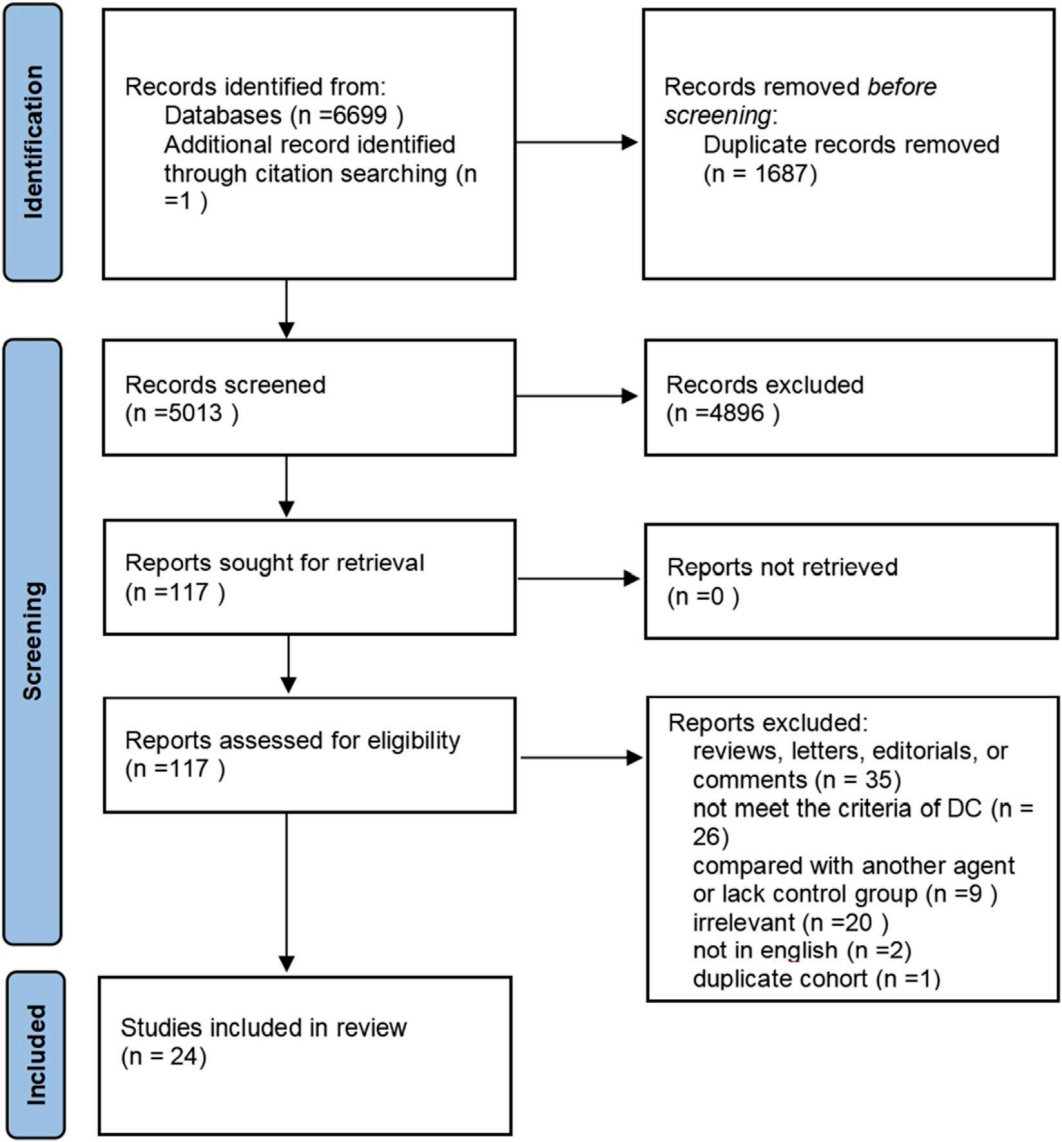
The literature screening flowchart. Decompensated cirrhosis, DC.

The systematic literature search identified 6,699 papers, with 1 additional record identified through citation searching. After removal of 1687 duplicates, 5,013 records were screened on the basis of titles and abstracts, of which 4,896 were excluded as clearly irrelevant. The remaining 117 full-text articles were assessed for eligibility. Of these, 93 were excluded for the following reasons:35 were reviews, letters, editorials, or comments; 26 did not meet the criteria of decompensated cirrhosis, 9 compared NSBBs with another agent or lacked a control group; 20 were irrelevant; 2 were not in English; and 1 represented a duplicate cohort. Ultimately, 24 studies (Sersté et al., 2010; Mandorfer et al., 2014; Bossen et al., 2016; Nawaz et al., 2026; Kang et al., 2021; Lee et al., 2020; Sersté et al., 2015; Bhutta et al., 2018; Leithead et al., 2015; Sinha et al., 2017; Tergast et al., 2019; Terres et al., 2022; Onali et al., 2017; Kimer et al., 2015; Aday et al., 2016; Snoga et al., 2020; Mookerjee et al., 2016; Fernández-Soro et al., 2026; Cholongitas et al., 2006; Bang et al., 2016; Sohn et al., 2020; Yoo et al., 2020; Chen et al., 2022; Wu et al., 2025) met the predefined inclusion criteria. The literature screening flowchart is depicted in Figure 1.

### 3.2 Study and patient characteristics

The characteristics of the 24 included studies are summarized in Table 1. The studies were published between 2006 and 2026 and included a total of 120,072 patients, of whom 110,632 (92.1%) received NSBBs and 9,440 (7.9%) did not. Sample sizes ranged from 56 to 23,602 patients per study. The cause of liver cirrhosis in most patients was alcoholic. Among the 24 studies, 13 studies were conducted in Europe (Sersté et al., 2010; Bossen et al., 2016; Sersté et al., 2015; Leithead et al., 2015; Sinha et al., 2017; Tergast et al., 2019; Onali et al., 2017; Kimer et al., 2015; Mookerjee et al., 2016; Fernández-Soro et al., 2026; Cholongitas et al., 2006; Bang et al., 2016),6 in Asia (Kang et al., 2021; Lee et al., 2020; Sohn et al., 2020; Yoo et al., 2020; Chen et al., 2022; Wu et al., 2025; Mandorfer et al., 2014), 4 in North American (Nawaz et al., 2026; Bhutta et al., 2018; Aday et al., 2016; Snoga et al., 2020), 1 in Brazil (Terres et al., 2022). Regarding study design, there were 1 RCT *post hoc* analysis of a randomized controlled trial (Bossen et al., 2016), and 23 cohort studies including 20 retrospective studies (Mandorfer et al., 2014; Nawaz et al., 2026; Kang et al., 2021; Lee et al., 2020; Sersté et al., 2015; Leithead et al., 2015; Sinha et al., 2017; Tergast et al., 2019; Terres et al., 2022; Onali et al., 2017; Kimer et al., 2015; Aday et al., 2016; Snoga et al., 2020; Fernández-Soro et al., 2026; Cholongitas et al., 2006; Bang et al., 2016; Sohn et al., 2020; Yoo et al., 2020; Chen et al., 2022; Wu et al., 2025) and 3 prospective studies (Sersté et al., 2010; Bhutta et al., 2018; Mookerjee et al., 2016). The most frequently used NSBB was propranolol, either alone (8 studies) (Sersté et al., 2010; Kang et al., 2021; Lee et al., 2020; Sersté et al., 2015; Cholongitas et al., 2006; Bang et al., 2016; Yoo et al., 2020; Chen et al., 2022) or in combination with carvedilol or nadolol (11 studies) (Mandorfer et al., 2014; Bossen et al., 2016; Nawaz et al., 2026; Bhutta et al., 2018; Leithead et al., 2015; Tergast et al., 2019; Onali et al., 2017; Snoga et al., 2020; Mookerjee et al., 2016; Fernández-Soro et al., 2026; Wu et al., 2025); 1 study (Kimer et al., 2015) used propranolol or metoprolol,1 used carvedilol exclusively (Sinha et al., 2017), and the other 3 studies (Terres et al., 2022; Aday et al., 2016; Sohn et al., 2020) did not specify the NSBB agent used.

**TABLE 1:** Characteristics of study.

First author and year	Country	Follow-up	NSBBs, n	Control, n	Type and dose of NSBBs, mg	NOS
Nawaz 2026	United States	At least 2y	104, 188	199, 914	Propranolol, nadolol or carvedilol, dose NR	9
Fernández-Soro 2026	Spain	150 m	47 (p 25, c 22)	52	Propranolol or carvedilol Dose p 80 mg c 12.5 mg	6
Wu 2025	China	5 y	58	184	Propranolol or carvedilol, dose NR	7
Chen 2022	China, taiwan	60 m	1788	1788	Propranolol, dose <80 mg n = 1532	8
Terres 2022	Brazil	12 m	21	25	NR	4
Kang 2021	Korea	NR	NR	NR	pp:Propranolol, dose NR	6
​	Korea	NR	NR	NR	Sp:Propranolol, dose NR	6
Yoo 2020	Korea	5 y	176	95	Propranolol, dose 40 mg	7
Sohn 2020	Korea	60 m	NR	NR	NR	5
Snoga 2020	United States	24 m	225	30	Propranolol or nadolol Media dose p 20 (20–40)mg n 30 (20–100)mg	5
Lee 2020	China, taiwan	5 y	650	1177	Propranolol, dose <30 mg 72.4%	9
Tergast 2019	Germany	28 d	255 (p 147, c 108)	369	Propranolol or carvedilol Median dose p 30 mg c 12.5 mg	7
Bhutta 2018	North American	375 d	307 (p 65, n 157, c 23)	411	Propranolol, nadolol or carvedilol Median dose p 40 mg n 40 mg c 12.5 mg	8
Sinha 2017	United Kingdom	5 y	132	193	Carvedilol, median dose 12.5 (range 6.25–12.5)mg	9
Onali 2017	United Kingdom	37 m	128 (p 118, c 10)	188	Propranolol or carvedilol Median dose p 80 mg c 6.25 mg	9
Mookerjee 2016	European	28 d	164 (p 111, n 6, c 16)	185	Propranolol, nadolol,carvedilol or other Median dose p 40 mg n 40 mg c 12.5 mg	8
Bossen 2016	Denmark	54 w	559	629	Propranolol or carvedilol Dose high-dose n = 159 low-dose n = 400	8
Bang 2016	Denmark	2 y	603	2472	Mildly decompensated cirrhosis Propranolol, median dose 97 (77–145)mg	9
​	Denmark	2 y	129	515	Severely decompensated cirrhosis Propranolol, median dose 96 (67–129)mg	9
Aday 2016	United States	NR	617	941	NR	6
Kimer 2015	Denmark	1500 d	23	38	Propranolol or metoprolol, media dose 80 (range 40–200)mg	5
Leithead 2015	United Kingdom	800 d	159 (p 119, c 40)	163	Propranolol or carvedilol Median dose p 80 (range 10–240)mg c 6.25 (3.125–12.5)mg	9
Serste 2015	Belgium	168 d	48	91	Propranolol, mean dose 83.3 mg	7
Mandorfer 2014	Austria	7 y	245 (p 178, c 67)	362	Propranolol or carvedilol Median dose p 40 (range 20–120)mg, c 12.5 (range 6.25–25)mg	7
Serste 2010	France	48 m	77	74	Propranolol, mean dose 113.25 (46.07)mg	6
Cholongitas 2006	Greece	NR	33	101	Propranolol, mean dose 39.04 (16.08)mg	5

p, propranolol; n, nadolol; c, carvedilol; NR, not report; y, year; m, month; d, day. pp, primary prophylaxis; sp, second prophylaxis.

The characteristics of patients are summarized in Table 2. The mean MELD score ranged from 11 to 28.9 across 14 studies (Jachs et al., 2021; Bhutta et al., 2018; Leithead et al., 2015; Sinha et al., 2017; Tergast et al., 2019; Kimer et al., 2015; Aday et al., 2016; Snoga et al., 2020; Mookerjee et al., 2016; Cholongitas et al., 2006; Yoo et al., 2020; Wu et al., 2025; Calès et al., 2021; Wang et al., 2024), but was not reported in 10 studies (Nawaz et al., 2026; Kang et al., 2021; Lee et al., 2020; Aday et al., 2016; Snoga et al., 2020; Cholongitas et al., 2006; Bang et al., 2016; Yoo et al., 2020; Chen et al., 2022; Wu et al., 2025). The MAP ranged from 77 to 87.4 in 9 studies (Mandorfer et al., 2014; Bossen et al., 2016; Sersté et al., 2015; Bhutta et al., 2018; Tergast et al., 2019; Onali et al., 2017; Snoga et al., 2020; Mookerjee et al., 2016; Sohn et al., 2020), while systolic blood pressure was reported in 1 study (Sersté et al., 2010),and MAP was not reported in the remaining 14 studies (Nawaz et al., 2026; Kang et al., 2021; Lee et al., 2020; Leithead et al., 2015; Sinha et al., 2017; Terres et al., 2022; Kimer et al., 2015; Aday et al., 2016; Fernández-Soro et al., 2026; Cholongitas et al., 2006; Bang et al., 2016; Yoo et al., 2020; Chen et al., 2022; Wu et al., 2025). Ascites was reported in 23 studies (Sersté et al., 2010; Mandorfer et al., 2014; Bossen et al., 2016; Nawaz et al., 2026; Kang et al., 2021; Lee et al., 2020; Sersté et al., 2015; Bhutta et al., 2018; Leithead et al., 2015; Sinha et al., 2017; Terres et al., 2022; Onali et al., 2017; Kimer et al., 2015; Aday et al., 2016; Snoga et al., 2020; Mookerjee et al., 2016; Fernández-Soro et al., 2026; Cholongitas et al., 2006; Bang et al., 2016; Sohn et al., 2020; Yoo et al., 2020; Chen et al., 2022; Wu et al., 2025), with prevalence ranging from 25.7% to 100%.12 studies (Mandorfer et al., 2014; Bossen et al., 2016; Lee et al., 2020; Bhutta et al., 2018; Sinha et al., 2017; Tergast et al., 2019; Terres et al., 2022; Onali et al., 2017; Mookerjee et al., 2016; Fernández-Soro et al., 2026; Cholongitas et al., 2006; Sohn et al., 2020) reported spontaneous peritonitis, with prevalence ranging from 3.7% to 100%.5 studies (Leithead et al., 2015; Tergast et al., 2019; Terres et al., 2022; Onali et al., 2017; Fernández-Soro et al., 2026) reported HRS or acute kidney injury (AKI), with prevalence ranging from 1.6% to 100%.10 studies (Sersté et al., 2010; Lee et al., 2020; Sersté et al., 2015; Terres et al., 2022; Onali et al., 2017; Snoga et al., 2020; Mookerjee et al., 2016; Fernández-Soro et al., 2026; Cholongitas et al., 2006; Sohn et al., 2020)reported HE, with prevalence ranging from 6% to 100%.

**TABLE 2:** Characteristics of cirrhosis.

First author and year	MELD score (N/C)	MAP, mmHg (N/C)	Most etiology of cirrhosis, % (N/C)	ascites, % (N/C)	SBP, % (N/C)	AKI or HRS, % (N/C)	HE, % (N/C)
Nawaz 2026	NR	NR	NR	100%	NR	NR	NR
Fernández-Soro 2026	14.0 (11.0–17.0)/12.0 (10.0–16.0)	NR	ALD:70.2%/82.7%	100%	19.1%/32.7%	12.8%/15.4%	27.7%/42.3%
Wu 2025	NR	NR	NR	100%	NR	NR	NR
Chen 2022	NR	NR	Hepatitis B:31.4%/32.1%	100%	NR	NR	NR
Terres 2022	All cohort:27.0 (7.0)	NR	ALD:76.1%	NR	34.80%	100%	56.5%
Kang 2021^pp^	NR	NR	NR	100%	NR	NR	NR
Kang 2021^sp^	NR	NR	NR	100%	NR	NR	NR
Yoo 2020	NR	NR	Non-viral:59.7%/52.6%	100%	NR	NR	NR
Sohn 2020	All cohort:20.6 (9.4)	All cohort:83.0 (14.6)	ALD:46%	100%	100%	NR	19.70%
Snoga 2020	NR	85.0 (15.2)/86.0 (13.0)	ALD:69.3%/70.0%	61.3%/73.3%	NR	NR	50.2%/73.3%
Lee 2020	NR	NR	Viral hepatitis:57.7%/59.9%	25.8%/25.7%	3.7%/5.9%	NR	100%
Tergast 2019	18.3 (7.9)/19.4 (8.1)	77.0 (15.0)/78.0 (16.0)	ALD:47%/47%	100%	23%/24%	29%/26%	NR
Bhutta 2018	20.0 (8.0)/20.0 (8.0)	86.0 (14.0)/86.0 (14.0)	ALD alone:31%/34%	100%	54%/48%	NR	NR
Sinha 2017	12.0 (9.0)/13.0 (9.0)	NR	ALD:71.2%/81.3%	100%	30.3%/17.6%	NR	NR
Onali 2017	14.0 (6.0)/15.0 (8.0)	80.0 (15.0)/86.0 (14.0)	ALD:37%/46%	100%	27.4%/16.8%	1.6%/4.4%	36%/28%
Mookerjee 2016	27.1 (7.6)/28.9 (7.4)	78.2 (13.3)/78.9 (12.7)	ALD alone:54.1%/62.4%	86.8%/87.9%	15.5%/17.3%	NR	49.6%/49.1%
Bossen 2016	12.0 (8.0–15.0)/11.0 (8.0–15.0)	83.0 (73.0–90)/85.0 (76.0–93.0)	ALD alone:56%/60%	100%	16%/14%	NR	NR
Bang 2016^mdc^	NR	NR	No viral hepatitis	100%	NR	NR	NR
Bang 2016^sdc^	NR	NR	No viral hepatitis	100%	NR	NR	NR
Aday 2016	NR	NR	NR	100%	NR	NR	NR
Kimer 2015	15.0 (13.0–19.0)/15.5 (12.0–19.0)	NR	ALD:78.2%/78.9%	100%	NR	NR	NR
Leithead 2015	16.0 (5.0)/17.0 (5.0)	NR	ALD:37.7%/38.0%	100%	NR	4.4%/4.9%	NR
Serste 2015	27.2 (6.7)/27.4 (8.1)	77.8 (3.5)/87.4 (4.9)	ALD 100%	56.3%/62.3%	NR	NR	58.3%/51.1%
Mandorfer 2014	17.2 (9.9)/17.8 (11.0)	84.4 (13.2)/86.2 (13.2)	ALD:59%/53%	100%	35.1%/26.5%	NR	NR
Serste 2010	18.8 (4.0)/18.9 (4.2)	SP; 103 (91–119)/123 (11–139)	ALD:46.8%/66.2%	100%	NR	NR	42.8%/32.4%
Cholongitas 2006	NR	NR	Hepatitis B:42%/34%	100%	18%/32%	NR	6%/8%

Meld, Model for End-stage Liver Disease; MAP, mean arterial pressure; SP, systolic pressure; SBP, spontaneous bacterial peritonitis; AKI, acute kidney injury; HRS, hepatorenal syndrome.

HE, hepatic encephalopathy; ALD, alcoholic liver disease; NR, not report; pp, primary prophylaxis; sp, second prophylaxis; mildly decompensated cirrhosis, mdc; severely decompensated cirrhosis, sdc; NSBBs, n; Control, c.

### 3.3 Study quality

The quality assessment results of the 24 included studies are summarized in Table 1. Of the 24 studies, 13 (54.2%) were rated as high quality (NOS ≥7 stars), 11 (45.8%) as moderate quality (NOS 4–6 stars), and none as low quality (NOS <4 stars). The median NOS score was 7 (range: 4–9).

### 3.4 Overall survival

**FIGURE 2:**
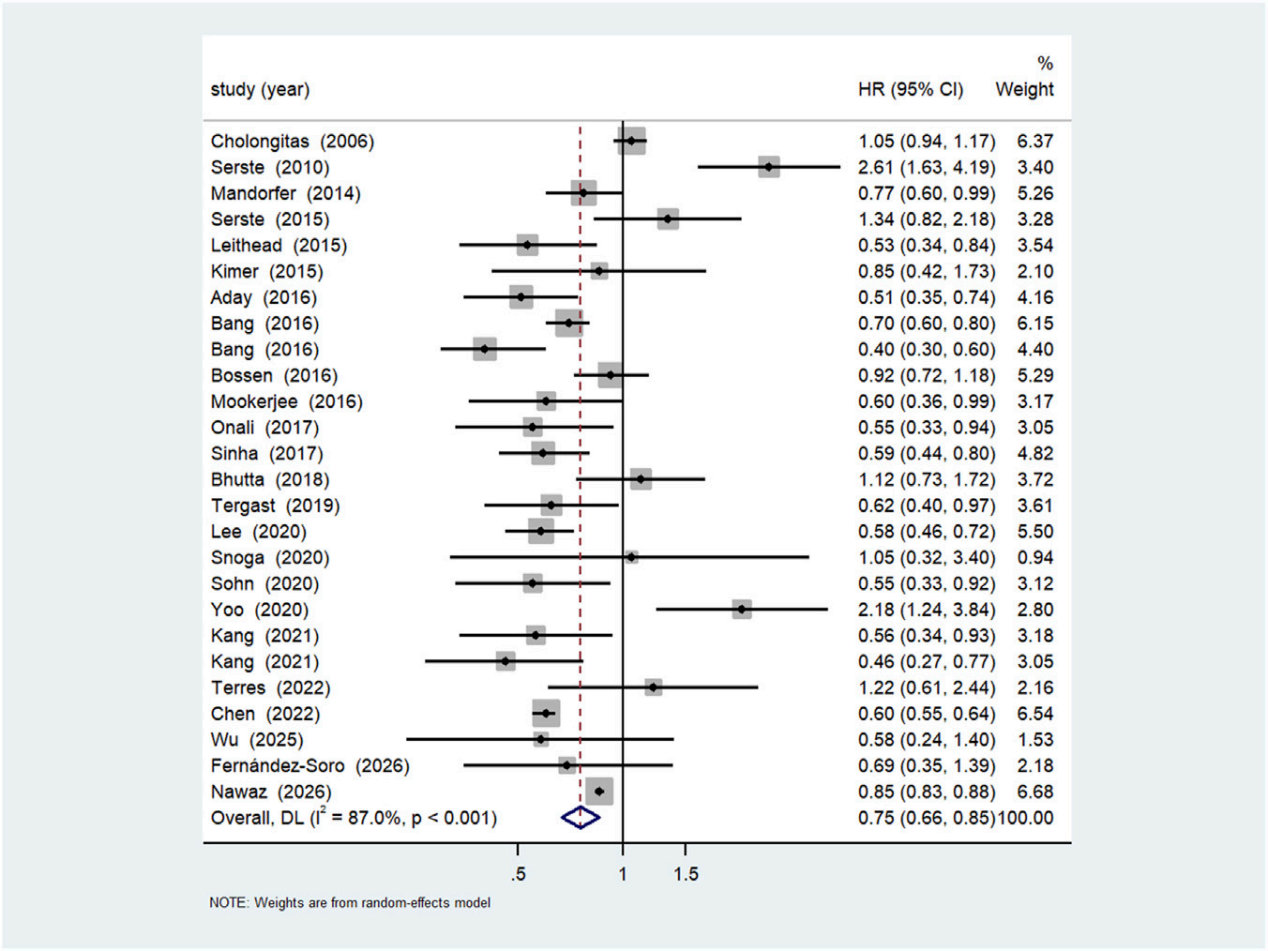
Forest plots of mortality risk associated with NSBBs in decompensated cirrhosis. Non-selective beta-blockers, NSBBs.

Meta-analysis of all 24 studies demonstrated that NSBB use was associated with a significant 25% reduction in mortality risk (HR = 0.75,95%CI = 0.66–0.85,p < 0.01; Figure 2). However, substantial heterogeneity was observed across studies (I^2^ = 87%,p < 0.01).

To assess the robustness of the result and investigate whether the statistical adjustment method influenced the pooled estimates, we performed a sensitivity analysis stratified by the analytical approach of the original studies.8 studies utilizing Propensity Score-Matched (PSM) (Nawaz et al., 2026; Lee et al., 2020; Leithead et al., 2015; Sinha et al., 2017; Onali et al., 2017; Bang et al., 2016; Yoo et al., 2020) demonstrated a consistent protective effect (HR = 0.72, 95%CI:0.59–0.88, p < 0.01; Supplementary Figure 1), while 13 cohorts (Sersté et al., 2010; Mandorfer et al., 2014; Bossen et al., 2016; Bhutta et al., 2018; Leithead et al., 2015; Tergast et al., 2019; Onali et al., 2017; Snoga et al., 2020; Mookerjee et al., 2016; Fernández-Soro et al., 2026; Bang et al., 2016; Chen et al., 2022) that employed multivariable regression analysis without PSM yielded a similar estimate (HR = 0.74,95%CI:0.61–0.89, p < 0.01; Supplementary Figure 2). The direction and magnitude of the effect were comparable, indicating that our primary result is robust.

### 3.5 Subgroup analysis

**FIGURE 3:**
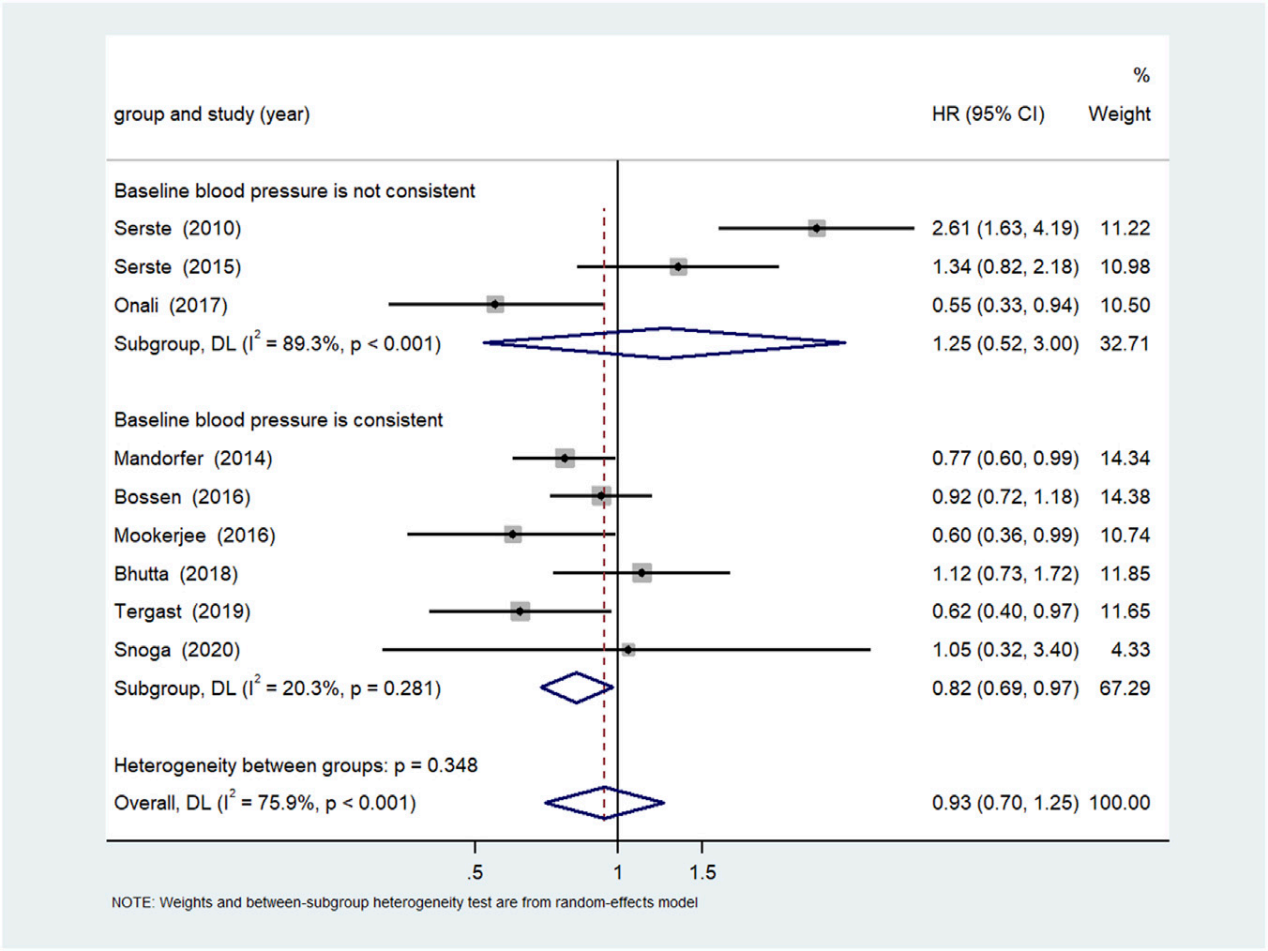
Forest plots of mortality risk associated with NSBBs in decompensated cirrhosis, stratified by blood pressure. Studies were classified as consistent baseline blood pressure if there was no statistically significant difference in baseline MAP or systolic pressure between groups, and as not consistent baseline blood pressure if a significant difference was present. Non-selective beta-blockers, NSBBs; MAP, mean arterial pressure.

The pooled analysis of 6 studies (Mandorfer et al., 2014; Bossen et al., 2016; Bhutta et al., 2018; Tergast et al., 2019; Snoga et al., 2020; Mookerjee et al., 2016) with consistent baseline blood pressure (BP) between NSBB and non-NSBB groups showed that NSBBs significantly reduced mortality risk (HR = 0.82, 95%CI:0.69–0.97, p = 0.02), with low heterogeneity (I^2^ = 20.3%,p = 0.28). In contrast, pooled analysis of 3 studies (Sersté et al., 2010; Sersté et al., 2015; Onali et al., 2017) with baseline BP imbalance between groups indicated no mortality benefit (HR = 1.25, 95% CI: 0.52–3.00, P = 0.62), with substantial heterogeneity (I^2^ = 89.3%, p < 0.01; Figure 3).

**FIGURE 4:**
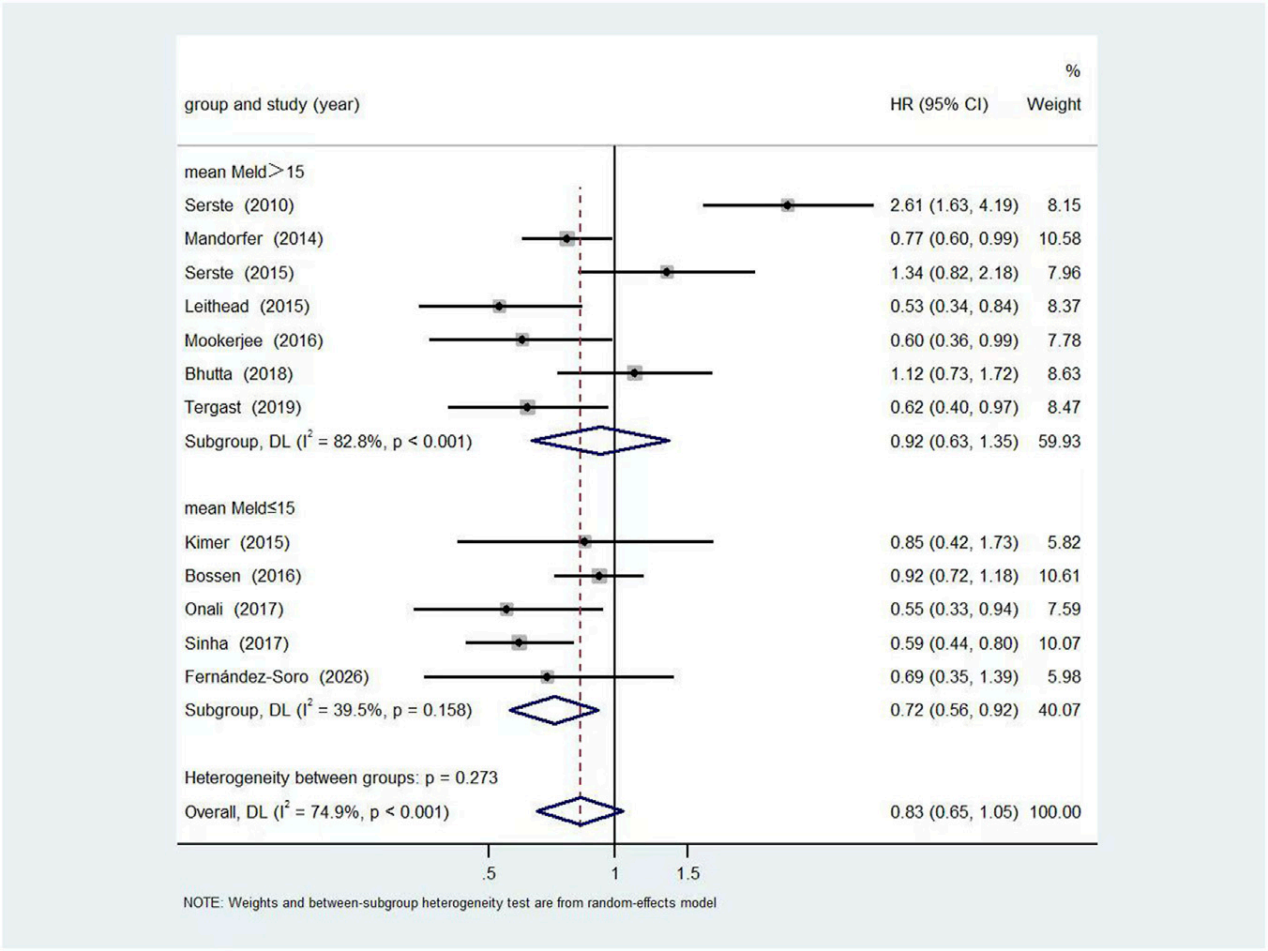
Forest plots of mortality risk associated with NSBBs in decompensated cirrhosis, stratified by MELD score. Non-selective beta-blockers, NSBBs; Model for end stage liver disease, Meld.

In 5 studies (Bossen et al., 2016; Sinha et al., 2017; Onali et al., 2017; Kimer et al., 2015; Fernández-Soro et al., 2026) with mean MELD scores of NSBBs cohort ≤15, NSBBs significantly reduced mortality (HR = 0.72, 95%CI:0.56–0.92, P < 0.01), with low heterogeneity (I^2^ = 39.5%, p = 0.16). In 7 studies (Sersté et al., 2010; Mandorfer et al., 2014; Sersté et al., 2015; Bhutta et al., 2018; Leithead et al., 2015; Tergast et al., 2019; Mookerjee et al., 2016) with mean MELD scores>15,NSBBs did not affect mortality risk (HR = 0.92,95%CI:0.63–1.35,P = 0.68),with substantial heterogeneity (I^2^ = 82.8%, p < 0.01; Figure 4).

**FIGURE 5:**
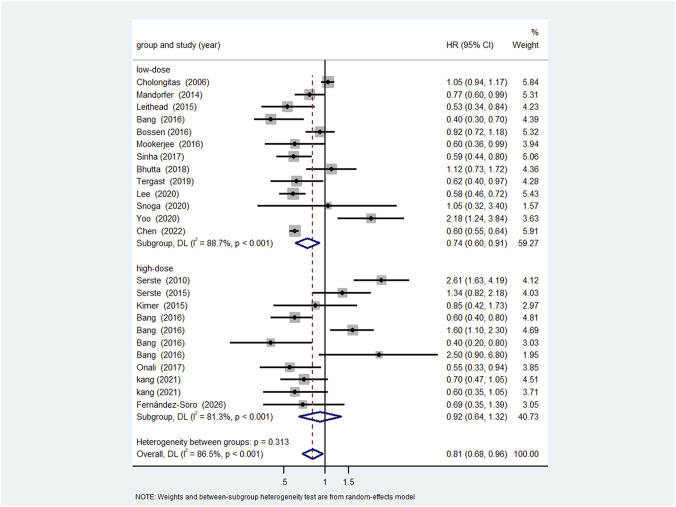
Forest plots of mortality risk associated with NSBBs in decompensated cirrhosis, stratified by dose. Studies were classified as low-dose if >50% of patients received low-dose NSBB, defined as propranolol or nadolol <80 mg daily, or carvedilol ≤12.5 mg daily; otherwise, high-dose. Non-selective beta-blockers, NSBBs.

A meta-analysis was conducted on the 13 studies (Mandorfer et al., 2014; Bossen et al., 2016; Lee et al., 2020; Bhutta et al., 2018; Leithead et al., 2015; Sinha et al., 2017; Tergast et al., 2019; Snoga et al., 2020; Mookerjee et al., 2016; Fernández-Soro et al., 2026; Bang et al., 2016; Yoo et al., 2020; Chen et al., 2022) evaluating low-dose NSBBs demonstrated a significant mortality reduction (HR = 0.74, 95%CI:0.60–0.91, p < 0.01), with substantial heterogeneity (I^2^ = 88.7%, p < 0.01). In 7 studies (Sersté et al., 2010; Kang et al., 2021; Sersté et al., 2015; Onali et al., 2017; Kimer et al., 2015; Fernández-Soro et al., 2026; Bang et al., 2016) evaluating high-dose NSBBs, no significant mortality benefit was observed (HR = 0.92,95%CI:0.64–1.32,P = 0.64), with substantial heterogeneity (I^2^ = 81.3%, p < 0.01; Figure 5).

19 studies (Sersté et al., 2010; Mandorfer et al., 2014; Bossen et al., 2016; Nawaz et al., 2026; Kang et al., 2021; Bhutta et al., 2018; Leithead et al., 2015; Sinha et al., 2017; Tergast et al., 2019; Onali et al., 2017; Kimer et al., 2015; Aday et al., 2016; Fernández-Soro et al., 2026; Cholongitas et al., 2006; Bang et al., 2016; Sohn et al., 2020; Yoo et al., 2020; Chen et al., 2022; Wu et al., 2025) that included patients with ascites demonstrated a significant mortality benefit with NSBBs (HR = 0.74, 95%CI:0.65–0.85, p < 0.01), and the heterogeneity was significant (I^2^ = 88.7%, p < 0.01; Supplementary Figure 3). 5 studies (Mandorfer et al., 2014; Nawaz et al., 2026; Tergast et al., 2019; Bang et al., 2016; Sohn et al., 2020) including patients with SBP showed NSBBs not influence the mortality risk of patients with decompensated cirrhosis (HR = 0.78, 95% CI: 0.55–1.11, P = 0.17; Supplementary Figure 4), with substantial heterogeneity (I^2^ = 80.4%,p < 0.01). 6 studies (Sersté et al., 2010; Mandorfer et al., 2014; Lee et al., 2020; Sersté et al., 2015; Snoga et al., 2020; Mookerjee et al., 2016) with HE prevalence >40% demonstrated that NSBBs did not affect the mortality risk of patients with decompensated cirrhosis (HR = 0.97, 95% CI:0.62–1.52, P = 0.90; Supplementary Figure 5), and the heterogeneity was significant (I^2^ = 86.8%, p < 0.01). Only one study (Terres et al., 2022) that combined HRS or AKI with a rate of more than 40% was available, so subgroup analysis could not be conducted.

### 3.6 Publication bias

Egger’s test was conducted to evaluate the presence of publication bias. The results indicated no significant publication bias in the studies (t = −0.96,P = 0.35).

## 4 Discussion

This meta-analysis synthesized data from 24 studies, encompassing over 100,000 patients with a follow-up from 28days to 5years. Our principal findings are twofold: (1) compared with the non-NSBB group, NSBB therapy was associated with a 25% reduction in mortality among patients with decompensated cirrhosis; however, this survival benefit was substantially influenced by MAP and the severity of liver disease; (2) low-dose NSBB regimens conferred a significant survival advantage in this population.

Our findings demonstrated a significant association between NSBBs use and reduced mortality in patients with decompensated cirrhosis, a result that was further substantiated by sensitivity analyses. The survival benefit conferred by NSBBs cannot be attributed solely to the reduction in portal pressure; it also arises from the modulation of non-hemodynamic parameters. Recently published data (Jachs et al., 2021; Tittanegro et al., 2022) indicated that NSBB therapy exert anti-inflammatory activity, as reflected by decreases in white blood cell (WBC) count, c-reactive protein (CRP), and interleukin-8 (IL-8) levels. In particular, Jachs et al., 2021 (Jachs et al., 2021) reported that an NSBB-induced reduction in WBC count of more than 15% is associated with lower mortality. Furthermore, NSBBs have been demonstrated to reduce intestinal barrier permeability and bacterial translocation (Jachs et al., 2021). Given that systemic inflammation driven by gut-derived bacterial translocation constitutes a core pathological mechanism of acute decompensation in cirrhosis (Arroyo et al., 2021; Kumar et al., 2024), NSBBs may attenuate this inflammatory cascade, thereby protecting against progression to more advanced cirrhosis and ultimately lowering mortality risk.

However, in patients with cirrhosis complicated by both SBP and HE, the survival benefit of NSBBs may be attenuated. Clearly, NSBBs are not universally beneficial across the entire spectrum of cirrhosis. Krag et al. proposed the “window hypothesis”, which posits that the safety and efficacy of NSBBs vary with disease stage, and that the therapeutic window of benefit may close once patients progress to end-stage liver disease (Albillos et al., 2023; Krag et al., 2012). The underlying mechanism arises from the hyperdynamic circulatory state characteristic of cirrhosis, in which elevated cardiac output and heart rate mediated by the sympathetic nervous system and renin-angiotensin system, serve as compensatory adaptations to splanchnic and systemic vasodilation. As cirrhosis advances, this hemodynamic cascade progressively compromises myocardial structure and function, manifesting as systolic and diastolic dysfunction (Boudabbous et al., 2024). These hemodynamic perturbations are clinically reflected by a decline in MAP in decompensated cirrhosis (Cullaro et al., 2024) and predispose patients to AKI (Cu et al., 2025). Lower MAP levels have been associated with a higher risk of AKI, further decompensation, and death in patients with cirrhosis (Cullaro et al., 2024). Moreover, AKI itself substantially increases the risk of mortality in cirrhotic patients with SBP (Sohn et al., 2020). In this hemodynamic context, the administration of NSBBs may further compromise systemic hemodynamics, thereby amplifying the risk of death. A retrospective study (Tergast et al., 2019) has demonstrated that in patients with SBP and a MAP <65 mmHg, NSBBs use is associated with renal impairment and confers no survival benefit, whereas in those with MAP ≥65 mmHg, NSBBs therapy does not precipitate renal injury and significantly improves 28-day survival. Similarly, a randomized controlled trial (Singh et al., 2022) revealed that propranolol was associated with worse survival compared with endoscopic variceal ligation (EVL) in patients with cirrhosis and grade≥2 ascites. The excess mortality risk observed in the study may be attributable to the finding that 57.5% of patients in the propranolol group had a MAP<82 mmHg, which likely provoked AKI. Giannelli et al., 2020 (Giannelli et al., 2020) further reported that among cirrhotic patients with refractory ascites, those with insufficient cardiac reserve defined as a left ventricular stroke work index <64.1 g m/m^2^ had an increased mortality risk when receiving NSBBs. Collectively, these findings indicate that MAP may serve as a critical determinant in guiding NSBB therapy in cirrhosis. Previous meta-analyses (Facciorusso et al., 2018; Wong et al., 2019) exhibited substantial heterogeneity and did not perform dedicated subgroup analyses in this specific population. In contrast, after accounting for baseline blood pressure in our meta-analysis, we found that NSBBs still confer a survival benefit in decompensated cirrhosis, with a concomitant and significant reduction in heterogeneity.

Similarly, the benefits of NSBBs in decompensated cirrhosis may be inversely correlated with the MELD score. A meta-analysis has demonstrated that the use of NSBBs in patients with cirrhosis and a MELD score >15 may exacerbate renal injury (Xu et al., 2024). Wang et al. (2024) also showed that NSBB use was significantly associated with an increased risk of further decompensation among patients with a MELD score >9. In addition, Calès et al. (2021) identified the baseline MELD score as an independent predictor of the deleterious effects of NSBBs on survival in cirrhosis. We therefore stratified our analysis according to the MELD score and found that the mortality risk associated with NSBB therapy varied across MELD strata. Specifically, the impact of NSBBs on mortality is attenuated in cirrhotic patients when the MELD score >15.

The dosage of NSBBs may influence prognosis in decompensated cirrhosis. A recent study (Kumar et al., 2024) showed that a median NSBB dose of 40 mg/day was associated with improved 28- and 90-day mortality in critically ill patients with cirrhosis. Madsen et al. (2016) also demonstrated a relationship between NSBB dose and mortality in patients with SBP, where low-dose therapy (80 mg) was linked to reduced mortality risk, whereas high-dose therapy (160 mg) trended toward increased risk. Furthermore, a real-world multicenter study also observed that, among patients with CTP class B/C, low-dose NSBBs conferred a 60% reduction in mortality risk, compared with only a 39% reduction with high-dose therapy (Kang et al., 2021). A recent randomized controlled trial (Kalambokis et al., 2021) further revealed that carvedilol at a dose of 12.5 mg/day may be the preferred NSBB treatment in patients with cirrhosis and ascites, as it improves renal perfusion and clinical outcomes. Consistent with these findings, our study demonstrated that low-dose NSBBs improved survival in patients with decompensated cirrhosis, whereas this benefit was attenuated with high-dose therapy. The underlying mechanism may involve a dual effect: low-dose NSBBs confer systemic anti-inflammatory benefits without compromising hemodynamic stability, whereas high-dose regimens tend to aggravate hemodynamic disturbances (Kumar et al., 2024).

This study has several limitations. A major limitation inherent to all included observational studies is the potential for residual confounding despite our use of multivariable regression and PSM. Furthermore, the association between NSBB use and mortality may be modulated by cirrhosis etiology (Ye et al., 2022), cardiac output (Alvarado-Tapias et al., 2020), and the specific agent administered (Kalambokis et al., 2021)—yet these factors could not be adequately explored. In our dataset, alcoholic liver disease accounted for the vast majority of cases, which reduced etiological heterogeneity; however, data on drug type and cardiac output were almost entirely absent across studies, thereby precluding any robust subgroup analyses. Second, although subgroup analyses stratified by MAP and MELD score partly explained the observed heterogeneity, considerable residual heterogeneity remained suggesting that other unmeasured factors may also contribute to the variability in treatment effects. Additionally, although MAP has been shown to influence outcomes in NSBB-treated patients, we were unable to perform a formal subgroup analysis stratified by a unified MAP cutoff value due to the fact that studies had MAP values within the range (77–87.4 mmHg), preventing a formal subgroup analysis based on a unified numerical cutoff. Final, while we additionally explored the impact of drug dosage on mortality, most studies lacked data on dose titrations and treatment discontinuation during follow-up, which may also have affected the observed associations.

## 5 Conclusion

Decompensated cirrhosis should not be regarded as an absolute contraindication to NSBB therapy; rather, the decision to initiate treatment, particularly at a low dose, may be guided by MAP and MELD scores. Nonetheless, these findings require further validation through randomized controlled trials.

## Data Availability

The original contributions presented in the study are included in the article/Supplementary Material, further inquiries can be directed to the corresponding author.
